# Supplementary material for: A genetically encoded sensor for visualizing leukotriene B4 gradients in vivo
Source: Nat Commun. 2023 Aug 1;14:4610. doi: 10.1038/s41467-023-40326-6 (PMC10393954; doi:10.1038/s41467-023-40326-6)
Supplement: Supplementary file 1 — Supplementary Information [file 41467_2023_40326_MOESM1_ESM.pdf]

Supplementary Fig.1

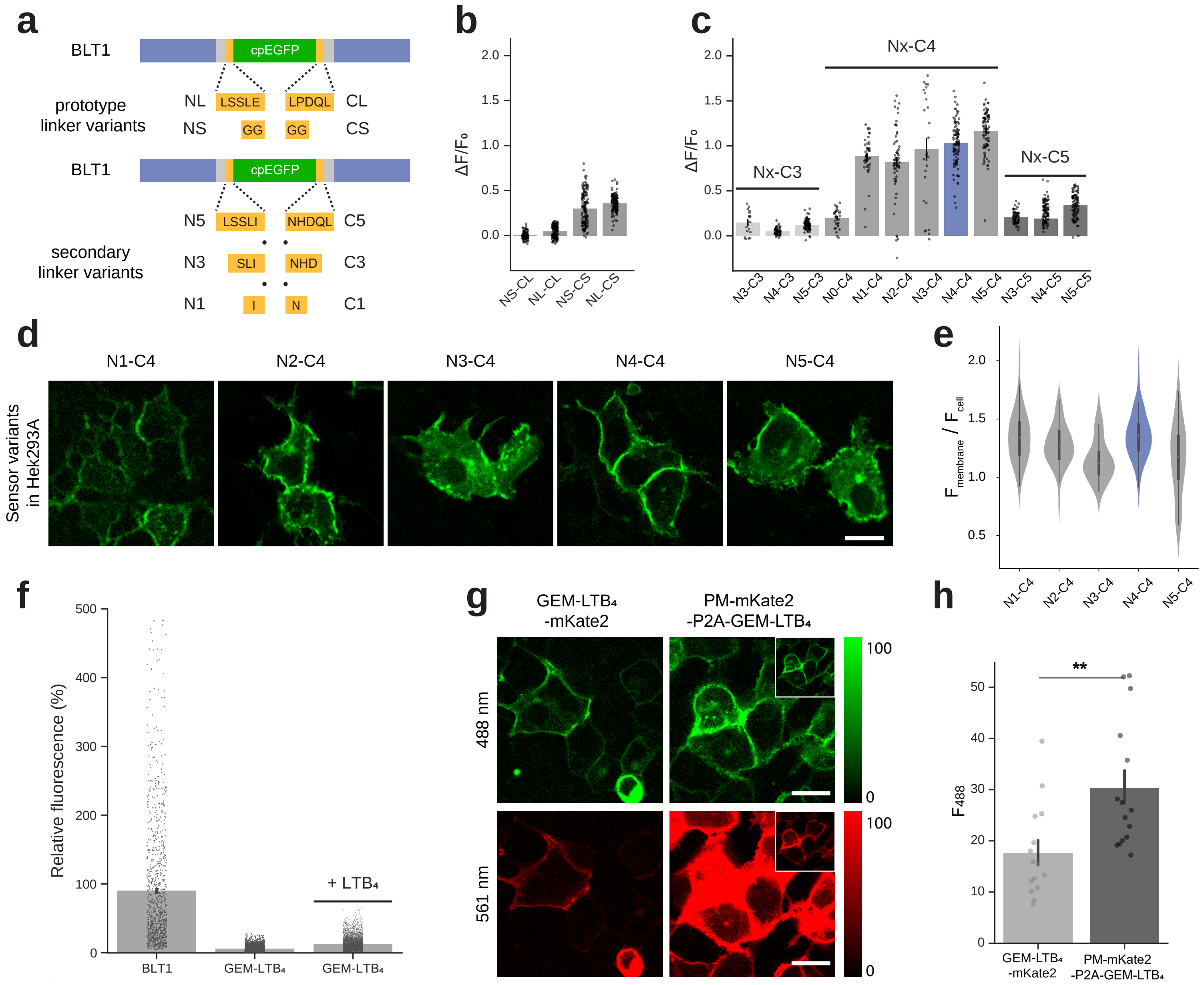

Supplementary Fig.1 | Linker length optimization of LTB_4 sensors.

a Schematic diagram of LTB_4-sensor design showing different N and C terminal linker variants used to create sensor prototypes. **b-c** Summary of $\Delta F/F_0$ fluorescence responses in sensor variants, shown as mean ± SEM, containing (**b**) prototype linker variants (n=106,134,135 and 139 cells for from 3 independent experiments) and (**c**) secondary linker variants (n=17, 61, 90, 30, 47, 62, 26, 97, 86, 62, 105 and 98 cells from 3 independent experiments). **d** Representative confocal fluorescence images of LTB_4-sensor expression in HEK293A cells with various indicated linker combinations. Scale bar, 25 µm. **e** Quantification of relative membrane localization of LTB_4-sensors shown in d, as $F_{membrane}/F_{cell}$ ratio expressed as mean ± SEM (n=54, 51, 23, 57 and 55 cells from 3 independent experiments). GEM-LTB_4 is shown in blue. **f** Relative GEM-LTB_4 fluorescence intensity in HEK293A cells with or without 100 nM LTB_4, compared to C-terminally GFP-tagged BLT1. Data shown as mean ± SEM (n=1010-900 cells from 3 independent experiments). **g** Representative confocal fluorescence images of HEK293A cells expressing GEM-LTB_4 with mKate2 directly fused to the C-terminus or co-expressed via the P2A self-cleavable peptide. Scale bars, 25 µm. **h** Quantification of average F_{488} intensities of GEM-LTB_4-mKate2 fusion variants shown in a, expressed as mean ± SEM (n=15 cells from 3 independent experiment, two-sided unpaired t-test, ***P*=0.003331).

Supplementary Fig. 2

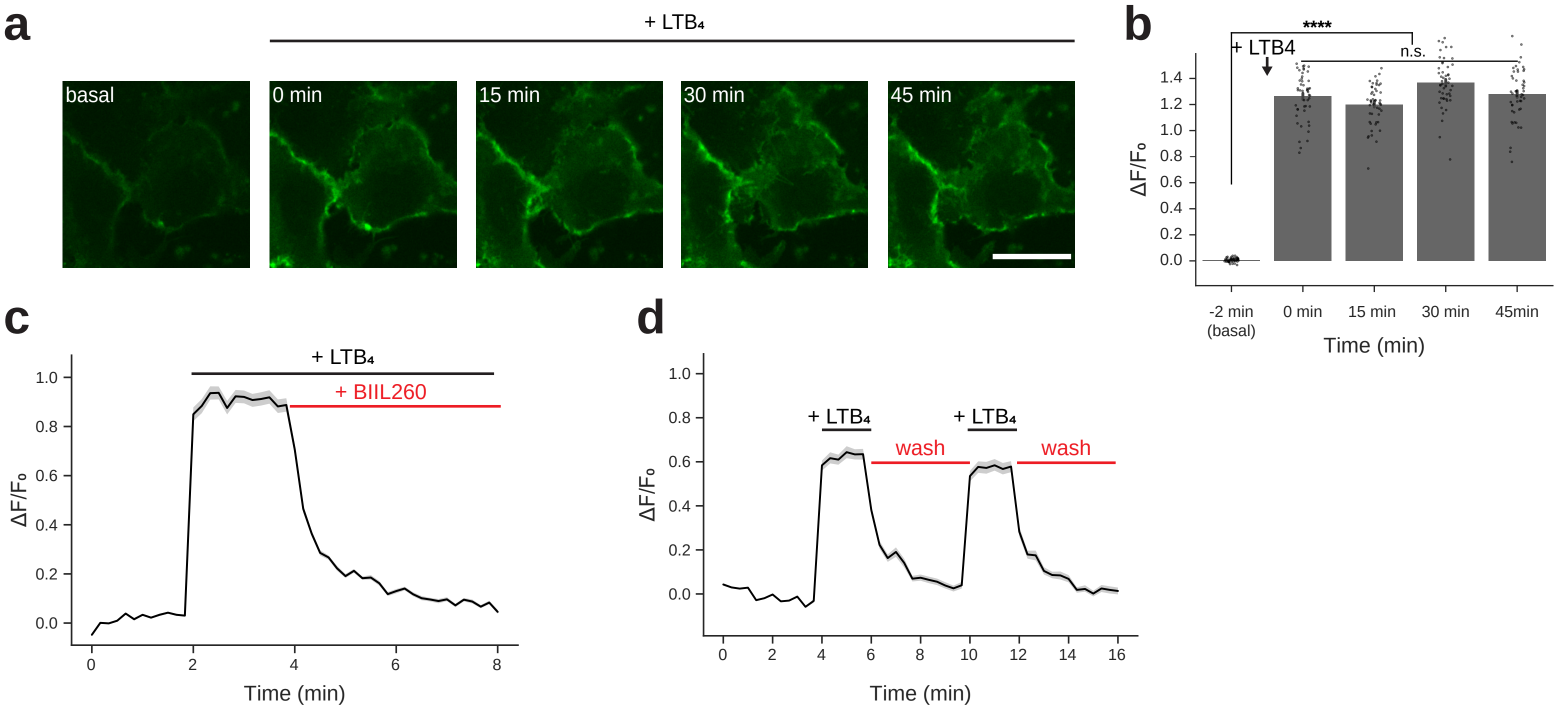

Supplementary Fig.2 | GEM-LTB$_4$ signals are sustained and reversible

a Representative GEM-LTB$_4$ confocal fluorescence time-lapse images of HEK293A cells over the course of a 45 min stimulation with 100 nM LTB$_4$. Scale bar, 25 µm. **b** Average $\Delta F/F_0$ fluorescence response of GEM-LTB$_4$, shown as mean ± SEM, during a 45 min stimulation with 100 nM LTB$_4$ (n=49 cells from 3 independent experiments). Statistical analysis was performed with one way repeated measure ANOVA (F=679.724, P=0.0) with Bonferroni correction (basal vs. 0-45 min: ****P=5.74*10^{-70}, 5.13*10^{-73}, 2.68*10^{-71} and 5.91*10^{-66}, respectively; and 0 min vs. 15-45 min: n.s., not significant, P=0.69, 0.055 and 1, respectively). **c** GEM-LTB$_4$ response to 100 nM LTB$_4$ stimulation followed by treatment with 1 µM of the BLT1 inhibitor BIIL-260. Data shown as mean ± SEM for n=83 cells from 3 independent experiments. **d** Average $\Delta F/F_0$ fluorescence response of GEM-LTB$_4$ during repetitive stimulation and wash cycles with 100 nM LTB$_4$. Data shown as mean ± SEM for n=344 cells from 3 independent experiments.

Supplementary Fig.3

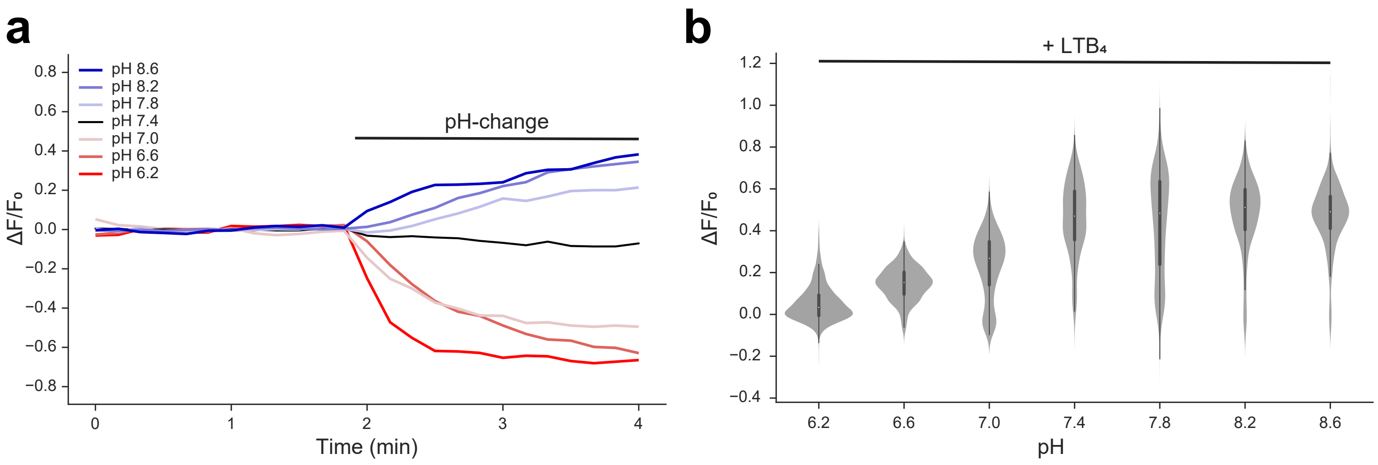

Supplementary Fig.3 | The effect of pH on GEM-LTB$_4$ signals

a Average traces of $\Delta F/F_0$ responses of GEM-LTB$_4$ expressing cells before and after perfusion of different pH-adjusted intracellular buffers (IC), supplemented with nigericin/monensin. Data shown as mean ± SEM for n= 226-596 cells from 3 independent experiments. **b** Average $\Delta F/F_0$ responses of GEM-LTB$_4$ expressing cells, directly after LTB$_4$ stimulation (100 nM) in different pH-adjusted IC buffers, supplemented with nigericin/monensin. Data shown as mean ± SEM for n=529-1256 cells from 3 independent experiments.

Supplementary Fig. 4

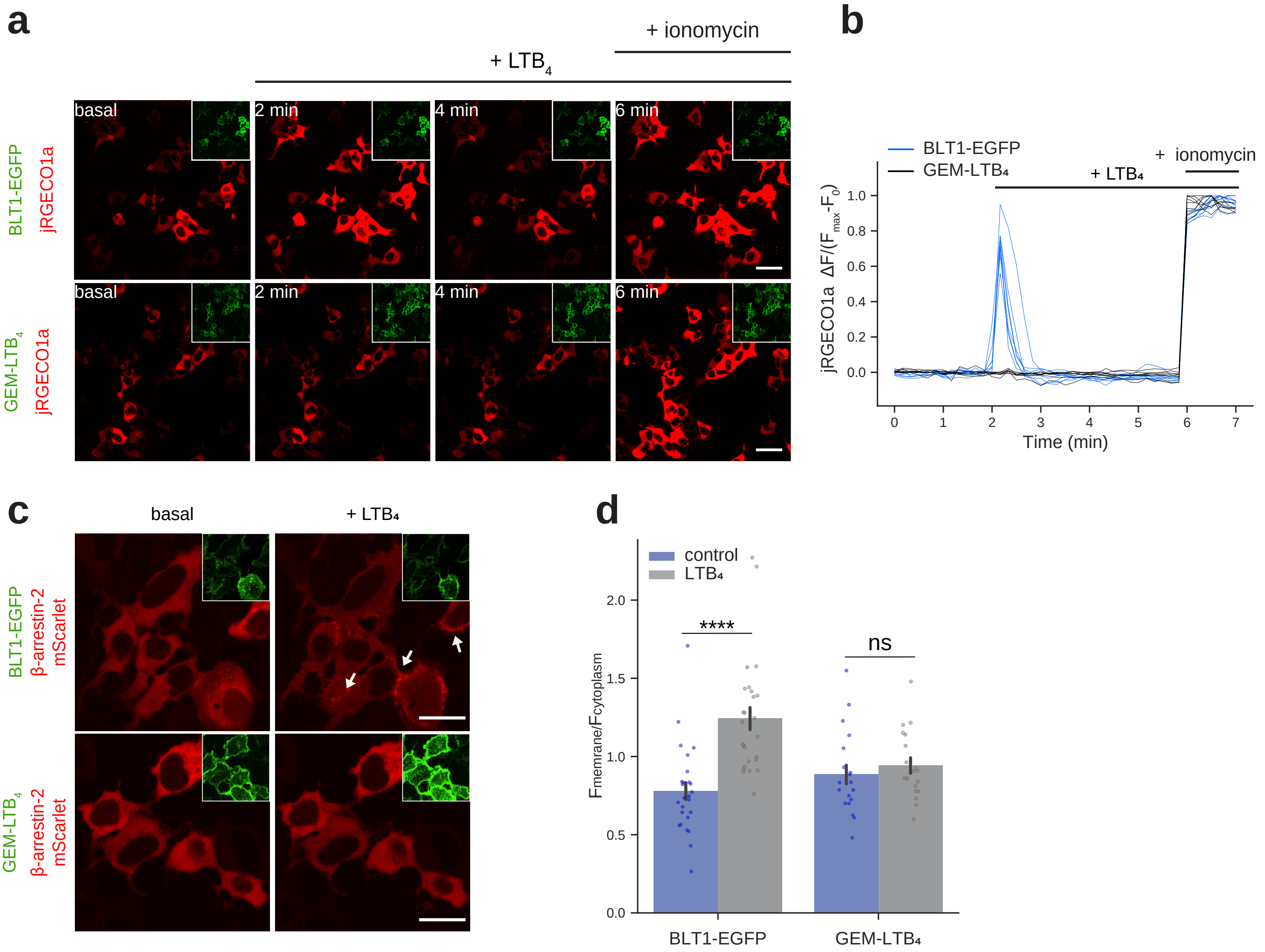

Supplementary Fig.4 | GEM-LTB_4 does not trigger GPCR signaling

a Representative confocal fluorescence images of the calcium sensor jRGECO1a (red) and BLT1-EGFP or GEM-LTB_4 (green) co-expressing HEK293A cells before (basal) and after 100 nM LTB_4 stimulus followed by treatment with 1 μM of the calcium ionophore ionomycin. Scale bars, 50 μm. **b** Individual traces of $\Delta F/(F_{max}-F_0)$ jRGECO1a responses in BLT1-EGFP or GEM-LTB_4 co-expressing cells, as shown in e, after 100 nM LTB_4 stimulus followed by stimulation with 1 μM ionomycin. Data shown as mean ± SEM for n=7 and 8 cells, respectively, from 3 independent experiments. **c** Representative confocal fluorescence images of β-arrestin-2-mScarlet (red) and BLT1-EGFP or GEM-LTB_4 (green) co-expressing HEK293A cells before (basal) and after 100 nM LTB_4 stimulus. Translocation of β-arrestin-2-mScarlet to the membrane is indicated by white arrows. Scale bar, 25 μm. **d** Quantification of relative β-arrestin-2-mScarlet translocation as a $F_{membrane}/F_{cytoplasm}$ ratio before and after 100 nM LTB_4 stimulus in BLT1-EGFP or GEM-LTB_4 co-expressing cells. Data shown as mean ± SEM for n=26 and 19 respectively from 2 independent experiments, with an two-sided unpaired t-test ****P=0.000003, n.s., not significant (P=0.058).

Supplementary Fig.5

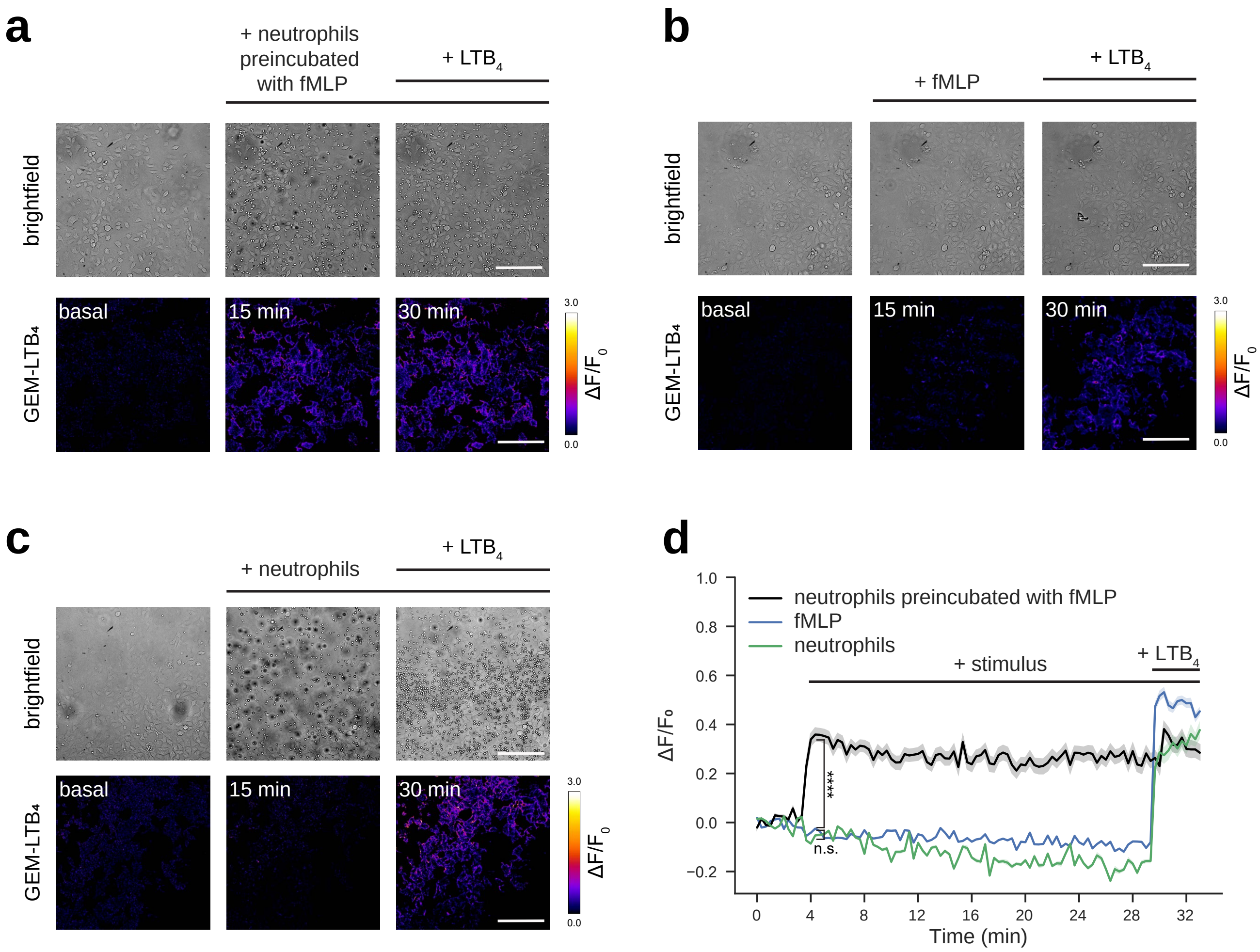

Supplementary Fig.5 | Measuring endogenous LTB_4 secretion from neutrophils with GEM-LTB_4

a-c Brightfield microscopy and corresponding $\Delta F/F_0$ ratio images of GEM-LTB_4 expressing HEK293A cells stimulated at 4 min with murine neutrophils ($2*10^6$ cells/cm^2) pretreated with 1 µM fMLP for 30 min (**a**)**,** with 1 µM fMLP alone (**b**), or with non-treated neutrophils (**c**). All treatments were followed by a 100 nM LTB_4 stimulus at 30 min. Scale bars, 100 µm. **d** Average traces of $\Delta F/F_0$ responses of GEM-LTB_4 expressing cells shown in a, b and c with respective stimulation at 4 min. Data shown as mean ± SEM for n=42 (a), 44 (b) and 38 (c) cells from 3 independent experiments. Statistical analysis were performed with one-way ANOVA (F=222.9, ****P=$2.6*10^{-41}$) with Fisher's LSD correction (fMLP-activated neutrophils being different from the other 2 conditions).

Supplementary Fig.6

a **b**

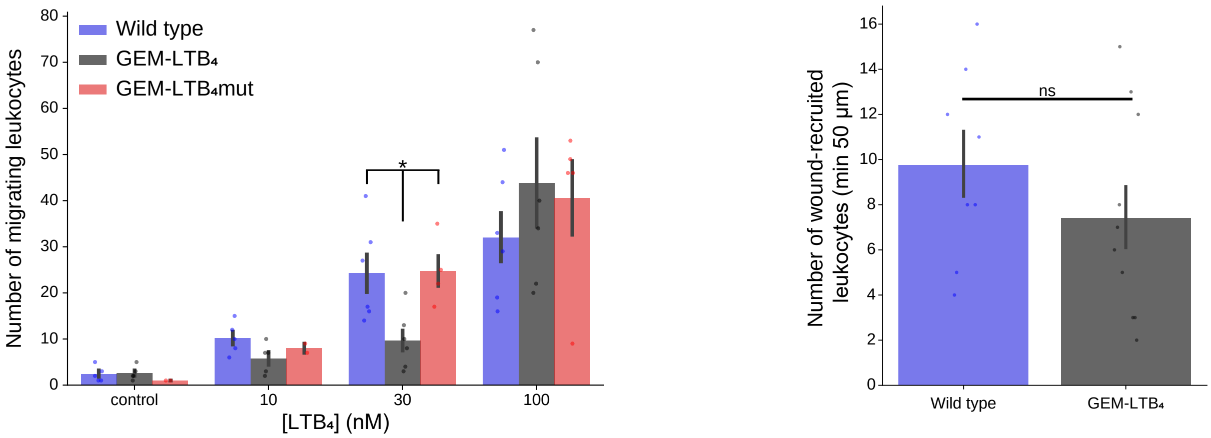

v	4.26 +/- 0.30 µm/min	3.66 +/- 0.30 µm/min
l	203.33 +/- 14.19 µm	154.76 +/- 17.56 µm
Dp	0.71 +/- 0.02	0.69 +/- 0.04
Dw	0.67 +/- 0.03	0.70 +/- 0.05

Supplementary Fig.6 | Exogenous and endogenous ligand buffering capacity of GEM-LTB$_4$ in zebrafish larvae

a Mean number of leukocytes migrating out of the notocord region of wild-type-, GEM-LTB$_4$- or GEM-LTB$_4$mut-expressing intact larvae stimulated with different doses of LTB$_4$ for 60 min. Data are presented as mean ± SEM for n=5-6 larvae for each condition from 3 independent experiments. Statistical analysis was performed separately on each dose with one-way ANOVA (control: F=0.849, n.s., not significant P=0.459; 10 nM: F=2.327, n.s. P=0.153; 30 nM: F=5.773, * P=0.016; 100 nM: F=0.604, n.s. P=0.56) with Fisher's LSD correction (30 nM: GEM-LTB$_4$ being different from the other 2 conditions). **b** Mean leukocyte recruitment within 60 min after tail fin transection of wild-type- or GEM-LTB$_4$-expressing larvae. Data are presented as mean ± SEM for n=8 and 11 larvae, respectively, from 3 independents experiments, with two-sided unpaired t-test (n.s., not significant, P=0.275).

Supplementary Fig. 7

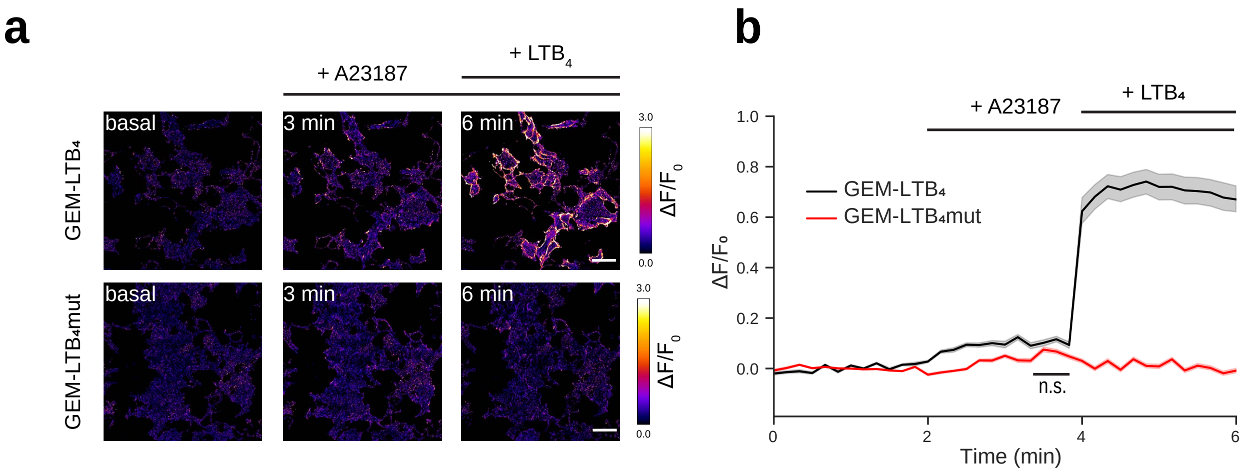

Supplementary Fig.7 | GEM-LTB$_4$ fluorescence is not affected by A23187

a Representative ΔF/F$_0$ images of GEM-LTB$_4$ and GEM-LTB$_4$mut expressing HEK293A cells treated with 1 μM A23187 followed by stimulation with 100 nM LTB$_4$. Scale bars, 50 μm. **b** Average traces of ΔF/F$_0$ responses in GEM-LTB$_4$ and GEM-LTB$_4$mut expressing cells shown in a. Data are presented as mean ± SEM for n=40 and 79 cells respectively from 3 independent experiments with two-sided unpaired t-test (n.s., not significant, *P*=0.09706) before LTB$_4$ stimulation**.**
