# Supplementary material for: A genetically encoded sensor for visualizing leukotriene B4 gradients in vivo
Source: Nat Commun. 2023 Aug 1;14:4610. doi: 10.1038/s41467-023-40326-6 (PMC10393954; doi:10.1038/s41467-023-40326-6)
Supplement: Supplementary file 3 — Description of Additional Supplementary Files Document [file 41467_2023_40326_MOESM3_ESM.pdf]

Description of Additional Supplementary Files Document

Supplementary Video 1 | Characterization of the GEM-LTB4 sensor

a, Fluorescence responses of GEM-LTB4 and GEM-LTB4mut to sequentially added increasing doses of LTB4 in HEK293A cells. b, GEM-LTB4 response to 100 nM LTB4 stimulation followed by treatment with 1 µM of the BLT1 inhibitor Cp105,696 or BIIL-260. c, Fluorescence responses of the calcium sensor jRGECO1a and BLT1-EGFP or GEM-LTB4 coexpressing HEK293A cells after 100 nM LTB4 stimulus followed by treatment with 1 µM ionomycin. d, β-arrestin2-mScarlet translocation in BLT1-EGFP or GEM-LTB4 co-expressing HEK293A cells after 100 nM LTB4 stimulus.

Supplementary Video 2 | Real-time measurement of LTB4 release from neutrophils with GEM-LTB4

a, GEM-LTB4 and GEM-LTB4mut responses in HEK293A cells after application of neutrophils followed by stimulation with 2 µM fMLP. Note the spreading increase of the green GEM-LTB4 signal followed by further increase from the 100 nM LTB4 stimulus. b, GEM-LTB4 responses (raw and normalized fluorescence) triggered by fMLP-activated neutrophils. Note the focal LTB4 production (middle and left panels) below a neutrophil (visible in the brightfield channel), and the lamellipodial movements of the surrounding neutrophils (right).

Supplementary Video 3 | Measuring exogenous LTB4 penetration with GEM-LTB4 in zebrafish

The reaction of GEM-LTB4 and GEM-LTB4mut in superficial epithelial cells of intact (left) or amputated (middle and right) zebrafish larvae stimulated with 1 µM LTB4.

Supplementary Video 4 | GEM-LTB4 reveals endogenous LTB4 release from leukocytes in zebrafish

GEM-LTB4 responses in the basal epithelial cells of wounded zebrafish larvae with parallel brightfield images of leukocyte recruitment. The 6 hour time-lapse video starts ~20 min post wounding. Note the increase in GEM-LTB4 signal starting at 1:20. Representative video of a larva showing LTB4 production (seen in 3 of 10 individually imaged larvae).

Supplementary Video 5 | GEM-LTB4 reveals ionophore-triggered LTB4 release from leukocytes in zebrafish

a, Confocal time-lapse of neutrophil calcium dynamics in Tg(lys:GCaMP7s-NES-P2A-mKate2-NES) larvae mounted in hypotonic agarose and stimulated with 100 µM A23187 after wounding and pre-incubation with 20 µM arachidonic acid for 90 min in isotonic E3 embryo media. b, GEM-LTB4 (top and bottom) and GEM-LTB4mut (middle) responses, in basal epithelial cells of zebrafish larvae wounded and pre-incubated in isotonic E3 embryo media with 20 µM arachidonic acid for 90 min alone or in combination with 20 µM of the 5-lipoxygenase inhibitor zileuton (bottom), and then stimulated with 100 µM A23187 after mounting in hypotonic agarose. Note the increase in GEM-LTB4 signal starting at 40 min (top panel) and the presence of moving leukocytes at the same time (right, brightfield).
